# Evaluation of the endoscopic revision of dacryocystorhinostomy failure cases: a cohort study

**DOI:** 10.1097/MS9.0000000000001039

**Published:** 2023-07-15

**Authors:** Khadija El Bouhmadi, Myriam Loudghiri, Youssef Oukessou, Sami Rouadi, Redallah Abada, Mohamed Roubal, Mohamed Mahtar

**Affiliations:** Department of Otolaryngology, Head and Neck Surgery, Ibn Rochd University Hospital, Faculty of Medicine and Pharmacy, Hassan II University, Casablanca, Morocco

**Keywords:** dacryocystorhinostomy, endoscopic, epiphora, revision surgery

## Abstract

**Introduction:**

The dacryocystorhinostomy (DCR) procedure is linked to a high success rate; however, cases of tearing recurrence are not rare and should be managed efficiently. Thus, evaluating cases of DCR failure allows highlighting the factors significantly impacting the results in order to realize better controlled primary surgeries.

**Material and method:**

Twenty-eight patients were operated in our Otolaryngology Department for endoscopic revision of DCR failure between January 2019 and June 2022. Their clinical presentation, postoperative evolution, and findings of the primary and revision surgeries were assessed until the actual follow-up.

**Results:**

The first surgery was based on an external approach in 17 patients and the bicanalicular silicone tube intubation was kept for a mean of 4.25 months. The recurrence delay varied from 0.5 to 9 months. Revision surgery revealed synechia in 10 patients, a completely closed DCR ostium in 22 patients (78.57%) by mucosal scarring and granulation, and lacrimal sac fibrosis in 16 patients (57.14%). A significant correlation was found between maintenance of the silicone intubation tube greater than or equal to 3 months and lacrimal sac fibrosis (*P*=0.016<0.05).

**Conclusion:**

Thus, better controlled primary surgeries with optimal exposure, wild marsupialisation of the lacrimal sac and no longer systematic bicanalicular intubation which should be dedicated to difficult anatomies and canalicular affections should guarantee better functional results.

## Introduction

Highlights

- Cases of tearing recurrence after dacryocystorhinostomy are not rare and should be managed efficiently.
- Evaluating cases of dacryocystorhinostomy failure allows highlighting the factors significantly impacting the results to realize better controlled primary surgeries.
- Lacrimal sac fibrosis is significantly correlated to long-term maintenance of the silicone intubation.
- Bicanalicular intubation should be dedicated to difficult anatomies and canalicular affections.

Dacryocystorhinostomy (DCR) involves the creation of a functional pathway to tears by opening the nasolacrimal sac with marsupialisation of its mucosa into the nasal cavity. Indicated for clinically significant nasolacrimal obstruction with epiphora and potential recurrent episodes of dacryocystitis, endoscopic, and external DCR are considered the gold standard techniques for nononcologic distal acquired lacrimal disorders^1–3^.

The first records on attempts of epiphora and dacryocystitis treatment date back to 2250 BC in the Code of Hammurabi. The nasal approach of DCR was the first to be described by Caldwell in 1893. Then, the external approach, thought by Toti and Dupuy-Dutemps in the early 20th century as the precursor of its current version, became the treatment reference since it is an open surgery, following a codified and reproducible technique. However, with the evolution of nasal endoscopes and mechanical tools, the endoscopic nasal procedure gained in popularity as an efficient and standardized technique^2,4^.

The postoperative outcome is related to the high rate of success, all surgical approaches combined. However, cases of tearing recurrence are not rare and should be managed efficiently. Therefore, evaluating cases of DCR failure allows highlighting the factors significantly impacting the results in order to realize better controlled primary surgeries.

## Material and method

We evaluated 28 cases of DCR failure operated in our department for endoscopic revision between January 2019 and June 2022.

These patients underwent primary DCR through an external approach with skin incision and trans-cutaneous lacrimal sac dissection (by ophtalmologists) or an endoscopic approach (by otolaryngologists); DCR for unilateral chronic tearing. The primary outcome was functional failure defined as recurrence of the epiphora (more than MUNK 2 scale) with or without episodes of dacryocystitis.

Before scheduling revision surgery, reinforcement of medical treatment was proposed, with a short course (5 days) of oral corticosteroids (1 mg/kg/day) or their local application directly on the granuloma if present. Then, the intubation tube was removed earlier to avoid perpetuating the inflammatory mechanisms.

The endoscopic revision surgery started with an exploration using 0 degree endoscope. Corrective septoplasty was performed in cases of obstructive septal deviation. Previous osteotomies were regularized with the high speed burr and the Kerrison rongeur to enlarge the exposition of the sac until its marsupialization confirming adequate bone removal. Then, the lacrimal sac was widely open with an 11 blade and bicanalicular silicone intubation was placed, next to a silastic nasal sheet in cases of septal surgery.

We analyzed the patient’s related factors (age, presence of coexistent chronic rhinosinusitis), the initial clinical presentation (presence of tearing, recurrent episodes of dacryocystitis, mean consultation period), the particularities of the first surgical procedure (surgical approach, osteotomies, aspect of the lacrimal sac), the postoperative evolution (treatment adherence, maintenance of silicone intubation), and the delay before the recurrence. Then, we assessed prospectively the findings of the endoscopic revision surgery (presence of synechia, septal deviation, the status of the ostium and of the lacrimal sac) until the actual follow-up.

The parameters were considered significantly impacting the surgical outcome if the Fisher’s exact test *P*<0.05.

The work has been approved by the ethical committee of our department. All the patients gave their consent for the surgery and the follow-up leading to the results of this study. The paper was written meeting the strengthening the Reporting of cohort, cross-sectional and case-control studies in surgery (STROCSS) 2021 criteria and was registered under the number 8646 on the Research Registry^5^.

## Results

The median age of our patients was 49 years (24–80 years), with a sex ratio of 0.21 (5M/23F) and a ground of coexistent chronic rhinosinusitis in four patients (14.28%). The initial clinical presentation was made of chronic unilateral tearing, of the right eye in 65% of the cases, with recurrent episodes of dacryocystitis. The mean consultation period was 5.21 years (1–30 years).

**Figure 1:**
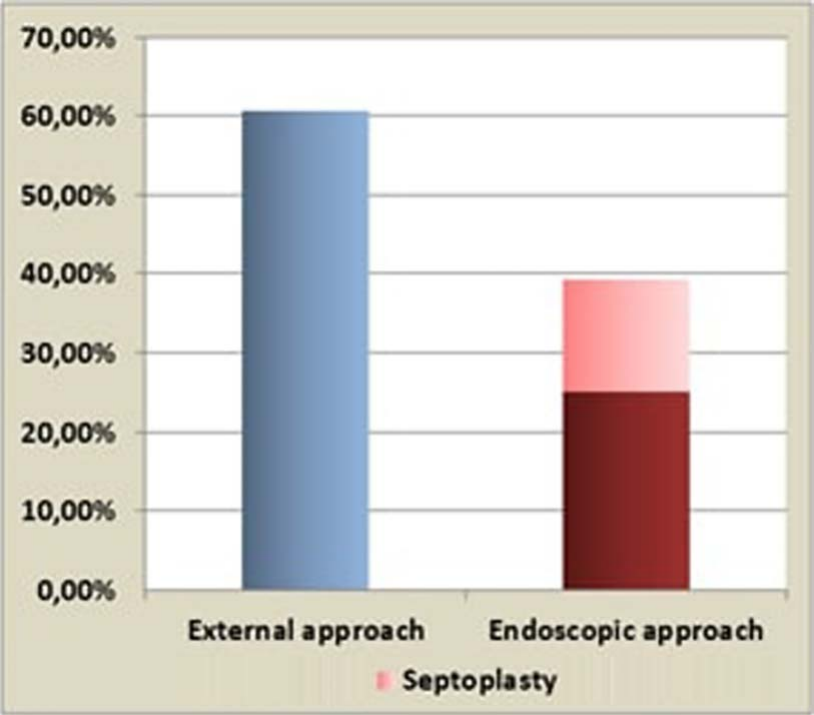
Different approaches for the first surgical procedure.

For the first surgical procedure, 17 patients were operated through an external approach; the other 11 underwent an endoscopic approach with concomitant septoplasty in four cases (Fig. 1). In these 11 patients, after removing the nasal mucosa overlying the desired area of bone removal, the osteotomies were realised using a combination of Kerrison rongeur and a high speed burr. Once, the lacrimal sac was exposed, its opening released pus and an intubation with a bicanalicular silicone tube was placed.

**Figure 2:**
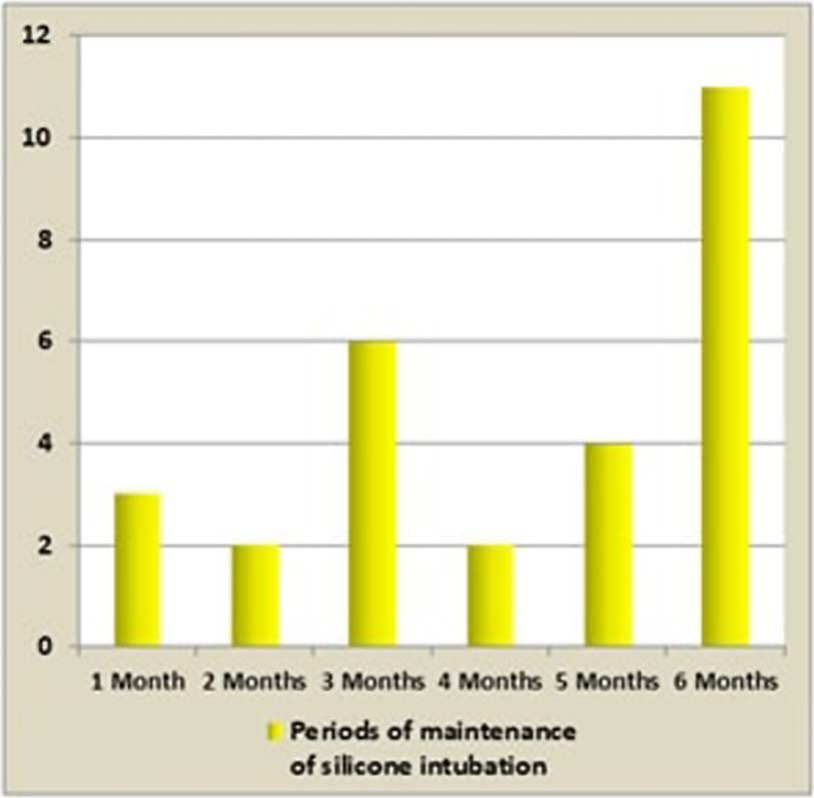
Periods of maintenance of silicone intubation.

Postoperatively, patients received oral antibiotherapy (amoxicillin/clavulanic acid) with nasal corticosteroids and nasal irrigation sprays and minor bleeding was observed in eight patients. The intubation tube was kept for a median period of 4.25 months (1–6 months, σ: 1.765) (Fig. 2). The mean time to recurrence of the epiphora was 2.47 months (0.5–9 months, σ: 2.155).

**Figure 3:**
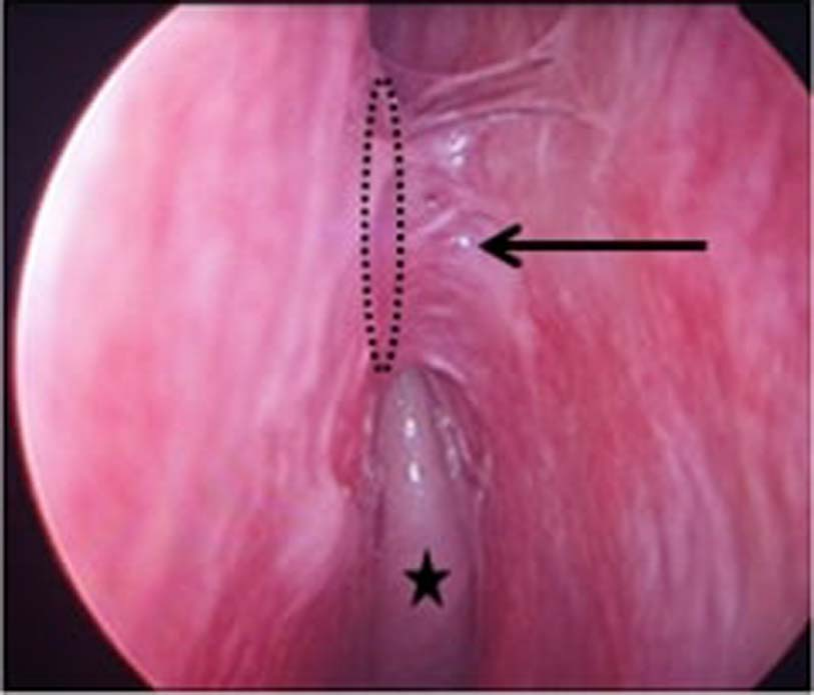
Obstructive synechia (black arrow) with the septum including the dacryocystorhinostomy ostium (completely closed and masked by the synechia, supposedly located in the doted circle) (black star: middle turbinate).

**Figure 4:**
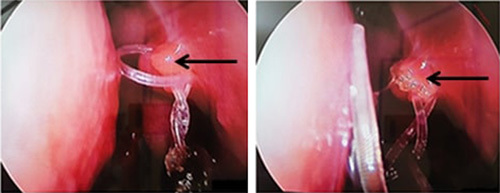
Granuloma (black arrow) totally closing the dacryocystorhinostomy ostium with the silicone tube within.

The mean period between the first surgery and the revision was 17.52 months (6–48 months, σ: 9.927). The surgical exploration, through an endoscopic approach, found obstructive synechia in 10 patients (Fig. 3) and septal deviation in five patients (four of them had previous external DCR), two of them needed corrective septoplasty. The ostium was narrowed in 21.42% of the cases and totally closed in 78.57% because of scar tissue growth in 18 patients and granuloma in four(Fig. 4).

In cases of insufficient bone removal, the previous osteotomies were enlarged with the high speed burr and the Kerrison rongeur to expose the totality of the sac until its marsupialization in the nasal fossa confirming adequate opening and exposure. At the opening of the lacrimal sac, pus was released in 11 patients (39.28%), fibrosis was found in 16 patients (57.14%), and a polyp in one patient (3.57%), which was sent to pathology analysis (Table 1). After widely opening the sac and exposing the common internal punctum, bicanalicular silicone intubation was placed, next to a silastic nasal sheet in cases of septal surgery.

**Table 1:** Findings of revision surgery of DCR failure according to the primary surgery approach.

		Primary surgery approach	
		External approach	Endoscopic approach
Findings of revision surgery			
Obstructive synechia	10 (43.47%)	6	4
Septal deviation	5 (17.85%)	4 Septoplasty in 2	1
Status of the ostium*Narrowed	6 (21.42%)	3	3
*Totally closed	22 (78.57%)		
Scar tissue growth	18 (81.81%)	10	8
Granuloma	4 (18.18%)	3	1
Lacrimal sac			
Pus release	11 (39.28%)	7	4
Fibrosis	16 (57.14%)	7	9
Polyp	1 (3.57%)	–	1

(*) indicates differenciate between the 2 options of the ostium status.

No significant incident was reported and postoperative follow-up did not observe any sign of recurrence until 40 months of control.

No significant correlation was found between the presence of lacrimal sac fibrosis and chronic rhinosinusitis ground (*P*=0.61>0.05) or the initial surgical approach (*P*=0.70>0.05). However, the maintenance of the silicone intubation tube greater than or equal to 3 months was significantly related to lacrimal sac fibrosis, which causes DCR failure (*P*=0.016<0.05) (Table 2).

**Table 2:** Evaluation of the correlation between lacrimal sac fibrosis and chronic rhinosinusitis, primary surgery approach, and the period of maintenance of silicone intubation.

	Lacrimal sac fibrosis		
Evaluated factors	Presence	Absence	*P*
Chronic rhinosinusitis			
Presence	3	1	0.61
Absence	13	11	
Primary surgery approach			
External	7	7	0.70
Endoscopic	9	5	
Maintenance of silicone intubation			
<3M	2	7	0.016<0.05
≥3M	14	5	

## Discussion

DCR involves the creation of a functional pathway from the canaliculi into the nose by means of creating an osteotomy and opening the nasolacrimal sac with marsupialisation of its mucosa into the nasal cavity. It can be performed via an external or endonasal approach and it is indicated for clinically significant nasolacrimal obstruction with epiphora and potential recurrent episodes of dacryocystitis^1,2^.

Postoperatively, antibiotics are not systematic and cleaning with nasal saline spray replaces instrumental removal of crusts. Nasal mucosa starts budding to rejoin lacrimal mucosa, which is more likely to retract than proliferate, until they merge generally at 2 months. Bertaux *et al.* ^6^ evaluated the evolution of the DCR ostium size by direct measurement with a surgical microhook every 2 months. They reported rapid and significant narrowing of the ostium in the 2 months following the removal of the intubation tube then, stabilization of its size after 6 months, with no correlation between the size reduction and the loss of function. However, a small ostium at 2 months postoperative was linked to a significant and early failure rate (100% at 6 months).

DCR is related to a high and comparable success rate consistently above 90% for external approach^7^ and ranging for from 82 to 98% for endoscopic approach^8^. However, cases of failure presenting with a recurrence of tearing may occur in a relatively short delay.

The endoscopic examination of the nasal cavity usually exposes the cause. Postoperative synechia are frequent after endoscopic DCR with mucosal trauma and they are only incriminated in surgical failure if obstructive, including the ostium. The ostium is often narrowed or closed by mucosal scarring, flat or with granulation, or by neo-osteogenesis. ‘Sump syndrome’ is described as a residual nasolacrimal sac, which collects fluids and leads to tearing^9^. And in some cases, no clear cause is observed when epiphora reappears after intubation tube removal despite permeable lacrimal ducts at washing and an open ostium of satisfactory size.

The comparison between the efficiency of the two DCR surgical approaches, external and endoscopic, hardly leads to unanimous consensus on the most suitable technique to the individual patient. Indeed, the treatment algorithm should be adapted and personalized according to the patient’s anatomy and condition. Vinciguerra *et al.* ^3^ review of the last 30 years of literature concluded that endoscopic DCR should be the reference treatment in revision cases or in patients with concomitant intranasal pathologies, while external DCR is indicated if local anesthesia is needed. If none of these conditions is present, the surgical approach is open to debate.

Indeed, the endonasal approach seems more suitable for revision as it allows better exploration of the nasal cavity, particularly when another concomitant nasal procedure is needed as in our study; while the external approach is to favor when canthal incision is necessary. More specifically, some authors observed in failed DCR cases more ostium narrowing in external DCR (*P*=0.043) and in laser DCR (*P*=0.047) than with an endonasal approach^10^.

Concerning the endoscopic approach, the literature reports overall better results when the uncinate process is removed than when the osteotomies are strictly maxillary. Also, another Vinciguerra *et al.* ^11^ review of the literature reported a mean success rate of 91.34% for DCR using different steel burrs and 89.5% when only cold instruments are used to dissect and open the lacrimal sac, with no significant difference between the surgical approaches (*P*=0.43).

Regarding the findings of revision surgeries, a better controlled primary surgery may prevent recurrence. It starts with better surgical exposure, mainly for endoscopic DCR, since enlarging the nasal cavity when needed avoid mucosal trauma. Septal deviation, even if considered uncomfortable for surgery only in 15% of the cases, should be corrected with minimally invasive endoscopic septoplasty with a silastic sheet, best if done simultaneously. Septoplasty replaces the temporary septal subluxation with Killian forceps, which presents the risk of fracturing the ethmoid cribriform plate in elderly patients. The middle turbinate can be partially removed in cases of Concha bullosa^2,12^.

The degree and regularity of bone and mucosal removal is important. Osteotomies should be large, better if removing the vertical part of the uncinate process until the lacrimal sac appears totally free and mobile in the surrounding bone secondary to external pressure^1,2,12^. Also, after large opening of the lacrimal sac in all its height, correct marsupialisation of the flaps should be performed, until they cover the osteotomy edges. Tissue glue can be used to ensure quick adhesion in the right position under visual control^2^. Usually, the mean success rate if mucosal flaps are used is 89 versus 92% if they are not used, with no statistical difference (*P*=0.14)^11^.

Intraoperative mitomycin C injection is linked to better results according to some authors, specifically in the difficult cases, with an optimal dose of (0.2 mg/ml), since it inhibits fibrous tissue growth, granulation, and scarring^2^.

Finally, no precise guidelines are described concerning peroperative bicanalicular silicone intubation. The actual consensus abandoned systematic intubation for an intubation if the surgeon considers its necessity. Also, its impact is still debated, while some authors report that it has no influence on the results, others consider it as a foreign body related to more narrowing and closing of the DCR ostium with a higher risk of granulation and scar tissue favouring the appearance of synechias, next to the risk of injury to lacrimal puncta. Thus, DCR with no silicone tube intubation should be the first choice of procedure, leaving the stenting for selected cases with poor local conditions preoperatively and intraoperatively assessed and canalicular affections (specific canalicular lesions such as inflammatory diseases like sarcoidosis or Wegener granulomatosis, tumors, or dacryolith)^1,11,13,14^.

Concerning the patient’s related factors, the literature does not report any influence of age and sex on the postoperative result of DCR, but the comorbid conditions increasing with age may affect the outcome, particularly eyelid laxity making necessary careful preoperative examination of the eyelid and conjunctiva^8^. Also, lower efficiency was observed in cases with nasal or palpebral chronic inflammatory pathologies such as blepharitis or sarcoidosis, suggesting postponing surgery until controlling the inflammatory affection^15^. Diabetes mellitus was also related to DCR failure since it predisposes to wound ulceration, granulation, and impaired healing by altering fibroblasts and endothelial cell functions producing granulation tissue and affecting proper vascularization of the wound^16^. On the other hand, it seems that surgical results were better when treating anatomical obstructions of the nasolacrimal duct system than functional ones^17^.

Our study follows the group of patients with DCR failure since their primary surgery to 40 months of follow-up after their endoscopic revision, evaluating the patients and the surgery related factors that may interfere with the outcome. Also, even with a low sample size, including patients primary operated through both external and endoscopic approaches globalize the findings. However, analyzing the results of revision surgery in each primary approach would enrich the literature data on the comparison between the two techniques.

## Conclusion

DCR is linked to a high rate of success, all surgical approaches combined. However, cases of tearing recurrence are not rare and should be managed efficiently. Thus, better controlled primary surgeries with optimal exposure, wide marsupialisation of the lacrimal should guarantee better functional results. Also, bicanalicular intubation should not be systematic. We propose to dedicate it to difficult anatomies and canalicular affections.

## Ethical approval

This work has been approved by the ethical committee of our department. All the patients gave their consent for the surgery and for the follow-up leading to this study.

## Consent

All the patients gave their consent for the surgery and for the follow-up leading to this study. Written informed consent was obtained from all the patients for publication of their results.

## Sources of funding

None.

## Author contribution

K.E.B.: study design, data collection and analysis and writing the paper; L.M.: contributor; Y.O.: study design; S.R.: study concept; R.L.A.: study concept; M.R.: correction of the paper; M.M.: correction of the paper.

## Conflicts of interest disclosure

The authors declare that they have no financial conflict of interest with regard to the content of this report.

## Research registration unique identification number (UIN)

researchregistry8646.

## Guarantor

Khadija El Bouhmadi.

## Provenance and peer review

Not commissioned, externally peer-reviewed.
